# Population bottleneck triggering millennial-scale morphospace shifts in endemic thermal-spring melanopsids

**DOI:** 10.1016/j.palaeo.2014.08.015

**Published:** 2014-11-15

**Authors:** Thomas A. Neubauer, Mathias Harzhauser, Elisavet Georgopoulou, Claudia Wrozyna

**Affiliations:** a Geological-Paleontological Department, Natural History Museum Vienna, Burgring 7, 1010 Vienna, Austria; b Institute for Earth Sciences (Geology and Paleontology), University of Graz, Heinrichstrasse 26, 8010 Graz, Austria

**Keywords:** Phenotypic evolution, Genetic drift, Morphometric analysis, Fast Fourier Transform, Stable isotopes

## Abstract

For more than hundred years the thermal spring-fed Lake Pețea near Oradea, Romania, was studied for its highly endemic subfossil and recent fauna and flora. One point of focus was the species lineage of the melanopsid gastropod *Microcolpia parreyssii*, which exhibited a tremendous diversity of shapes during the earlier Holocene. As a consequence many new species, subspecies, and variety-names have been introduced over time, trying to categorize this overwhelming variability. In contrast to the varied subfossil assemblage, only a single phenotype is present today. We critically review the apparent “speciation event” implied by the taxonomy, based on the presently available information and new data from morphometric analyses of shell outlines and oxygen and carbon isotope data. This synthesis shows that one turning point in morphological evolution coincides with high accumulation of peaty deposits during a short time interval of maximally a few thousand years. The formation of a small, highly eutrophic swamp with increased input of organic matter marginalized the melanopsids and reduced population size. The presented data make natural selection as the dominating force unlikely but rather indicates genetic drift following a bottleneck effect induced by the environmental changes. This claim contrasts the “obvious trend” and shows that great morphological variability has to be carefully and objectively evaluated in order to allow sound interpretations of the underlying mechanisms.

## Highlights

• Phenotypic diversity of a melanopsid species in a thermal spring is reviewed.
• Morphometric analyses and stable isotopes provide quantitative data.
• Environmental changes coincide with phenotypic shift during the early Holocene.
• Population decline caused a bottleneck triggering genetic drift.
• The absence of potential selective agents renders natural selection unlikely.

## 1 Introduction

**Fig. 1:**
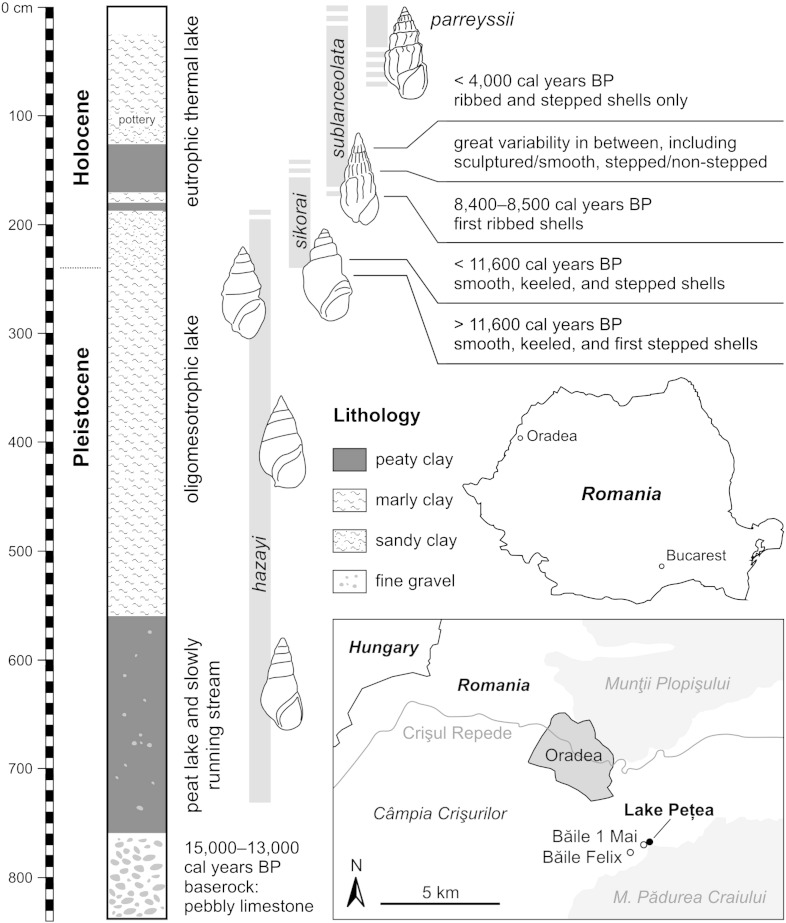
Geographic overview of the studied locality and section. The morphological succession and the phenotype names were adopted from Kormos (1905b). For a revised taxonomic concept see Chapter 5.6. and the Appendix. The dating as well as the paleoecological interpretation to the right of the section is correlated following the data of Sümegi et al. (2012b).

**Fig. 2:**
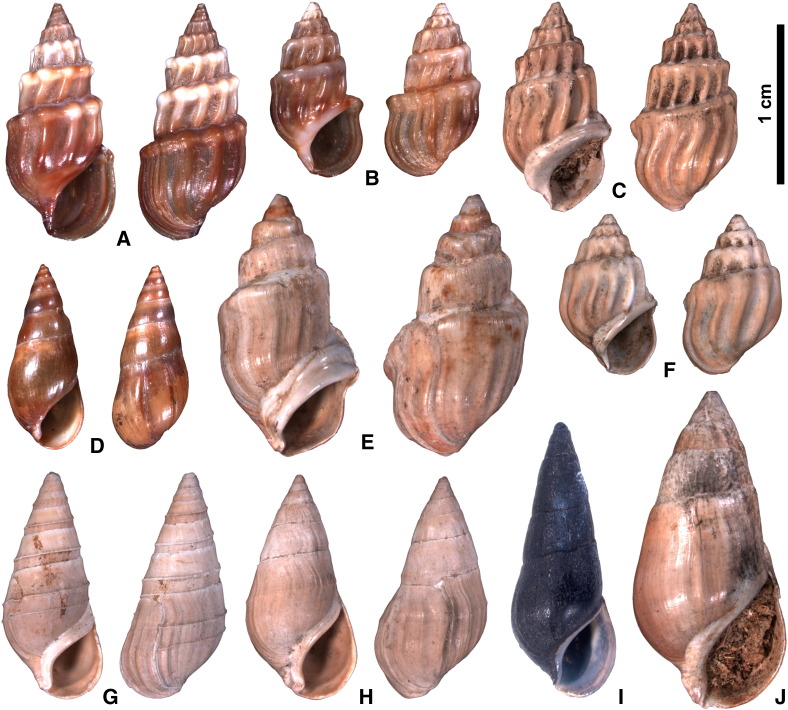
Several subspecies and phenotypes of *Microcolpia* illustrating their morphological variability. (A, B) *M*. *parreyssii parreyssii* (Philippi, 1847), recent, NHMW Moll. 109249, Lake Pețea, Romania. (C) *M*. *p*. *parreyssii*, NHMW 1908/0012/0041, Lake Pețea, Romania. (D) *M*. *daudebartii daudebartii* (Prevost, 1821), recent, NHMW Moll. 51381, Hansybach at Bad Vöslau, Austria. (E) *M*. *sikorai* sensu Brusina, 1903, NHMW 2013/0414/0002, Lake Pețea, Romania. (F) *M*. *p*. *parreyssii*, NHMW 1903/0001/0016, Lake Pețea, Romania. (G) *M*. *tothi* sensu Brusina, 1903, NHMW 2013/0414/0001, Lake Pețea, Romania. (H) *M*. *hazayi* sensu Brusina, 1903, NHMW 1989/0089/0113, Lake Pețea, Romania. (I) *M*. *daudebartii acicularis* (Férussac, 1823), recent, NHMW Moll. 31501, Pesnica brook near Moškanjci, Slovenia. (J) *M*. *vidovici* sensu Brusina, 1903, NHMW 1908/0012/0059, Lake Pețea, Romania.

*Microcolpia parreyssii* (Philippi, 1847) is a thermophilic melanopsid species presently restricted to a single locality, the small thermal spring-fed Lake Pețea (*Rom*. Băile 1 Mai, Băile Episcopale; *Hung*. Püspökfürdő; *Germ*. Bischofsbad; Fig. 1), situated about 9 km SE of Oradea in W Romania. It is a morphologically well-defined taxon comprising distinctly stepped and ribbed shells. The morphological history of this species, however, draws a completely different picture. Shells from late Pleistocene to Holocene deposits of the thermal spring exhibit an extreme variability and a distinctly wider range of morphologies as present in the lake today. This range includes smooth, slender, and elongate shapes, stepped and non-ribbed forms, slender and keeled phenotypes, as well as subfossil representatives of typical *M*. *parreyssii* (Brusina, 1903; Kormos, 1903, 1904, 1905a,b; Paucă, 1937; Sümegi et al., 2012a,b; Fig. 2). This led to the introduction of a large number of names, trying to categorize this vast variability (Brusina, 1903; Kormos, 1905b). In total, 43 species-, subspecies-, variation- and forma-names have been introduced since then. Still, the taxonomic concepts applied by Brusina (1903) and Kormos (1905b) are unfortunately not clear from their descriptions — and illustrations are available only for a few phenotypes. Moreover, the taxon delimitations of both authors diverge considerably. Brusina (1903) himself recognized the strong over-splitting and suggested alternatively a series of synonymizations. The fluent morphological transitions, however, make splittings as well as synonymizations highly subjective anyway.

It has been demonstrated that the many phenotypes do not equally occur within the same time intervals, but rather show morphological changes over time (Kormos, 1905b; Sümegi et al., 2012a,b). The available data suggest that the succession of phenotypes is coupled with changes in the paleoenvironment. In former times Lake Pețea, which originated during the late stages of the Würmian glaciation, had a much larger extent (Sümegi et al., 2012a). Up to present the lake has shrunken to a size of a few hundred square meters and temperature has dramatically declined, what has a severe impact on the rich fauna, including several species of fish (Telcean and Cupşa, 2013), amphibians (Covaciu-Marcov et al., 2003), and gastropods (e.g., Brusina, 1903; Kormos, 1905b; Harzhauser and Mandic, 2008; Fehér et al., 2009; Sîrbu et al., 2013). Some of these are entirely endemic to this small environment and are critically endangered (Sîrbu and Sárkány-Kiss, 2002; Fehér, 2011; Sîrbu et al., 2013).

The aim of this paper is an objective quantification of shell shapes to document and elucidate the morphological variability in the recent and past melanopsids of Lake Pețea by means of a morphometric analysis. Additional information is provided by stable isotope analyses to reconstruct paleo-water conditions. Together with existing data on lake's development a detailed picture of the evolutionary pattern and its causes is drawn.

## 2 Materials

The entire material derives from the collections of the Natural History Museum of Vienna. The subfossil Holocene specimens are stored in the Geological-Paleontological Department (coll. nos. NHMW 1903/0001, 1908/0012, 2013/0414), the recent specimens in the 3rd Zoological Department (Malacology section, referred to as NHMW Moll.). The whole material displays a compound of several independent collection surveys and fully covers the morphological variability described in the literature. Unfortunately, it was not possible to obtain material from stratified collections to provide information on the morphospace evolution through time. Only adult and subadult specimens were analyzed, which can be detected over the slightly thickened inner lip; juveniles were excluded from the analysis.

A total number of 327 specimens were analyzed (Table 1). As to the subfossil part, 245 *Microcolpia* specimens from Băile 1 Mai, Romania (= Püspökfürdő, Bischofsbad) were selected. To keep an objective position any preliminary attribution to a species, subspecies, or phenotype was avoided before the morphological analyses. However, in the discussion we refer to the ribbed forms (recent and subfossil) as *Microcolpia parreyssii parreyssii* (Philippi, 1847) and to the remaining subfossil ones as *Microcolpia parreyssii sikorai* (Brusina, 1903). Although we do not want to anticipate the taxonomic implications of the present work (see also Chapter 5.6. and Appendix), this approach noticeably eases communication and prevents confusion with the many old names.

From the recent population 32 specimens of *Microcolpia parreyssii*. *parreyssii* from Lake Pețea (NHMW Moll. 31902, 75000/E/26689, 109249, 109250) were used. For comparison, 25 recent specimens of each *Microcolpia daudebartii daudebartii* (Prevost, 1821) from Bad Vöslau thermal spring, Austria (NHMW Moll. 51381), and *Microcolpia daudebartii acicularis* (Férussac, 1823) from Pesnica brook near Moškanjci, Slovenia (NHMW Moll. 31501), were selected. The comparably high number of subfossil specimens was necessary to sufficiently cover the range of morphological plasticity (Table 1). The only underrepresented morphologies are “non-stepped and ribbed” and “stepped and keeled”, both of which combinations occur very seldom. 30 specimens of those used for morphological analyses were selected for the isotope analysis: 20 subfossil and 5 recent shells from Lake Pețea and 5 shells from Bad Vöslau.

The superspecific systematics of the involved taxa is very unstable and varies even across the recent publications (e.g., Glöer, 2002; De Jong, 2012; Welter-Schultes, 2012). The only genetic data treating some of the respective taxa are presented by Smoleń and Falniowski (2011). They indicate a very close relationship between “*Melanopsis parreyssii*” and “*Fagotia acicularis*” and a great genetic distance of both taxa to *Melanopsis costata*. “*F*. *acicularis*” is the type species of *Microcolpia* Bourguignat, 1884, which is treated as distinct genus after the latest systematic revision (Welter-Schultes, 2012). Although the systematic conclusions of Smoleń and Falniowski (2011) are incorrect, that *M*. *costata* has to be placed in genus other than *Melanopsis* (despite being the type species), they imply that “*M*. *parreyssii*” is closer related to *Microcolpia* than to *Melanopsis*. Accordingly we classify all the melanopsid taxa from Lake Pețea within *Microcolpia*. The taxonomic separation of *Microcolpia daudebartii acicularis* and *Microcolpia daudebartii daudebartii* is debatable. In most studies they are considered separate subspecies (e.g., Fischer and Müller, 1996; Fischer, 2002; Glöer, 2002; Glöer and Sîrbu, 2005; De Jong, 2012). Others treat it as subjective junior synonym of *M*. *daudebartii* (Reischütz, 1994; Welter-Schultes, 2012).

It would be tempting to incorporate information from molecular data on the recent populations into the present analysis, but unfortunately the data from Smoleń and Falniowski (2011) is the only published genetic data including *Microcolpia parreyssii* and is based on two specimens only.

## 3 Methods

### 3.1 Shape analysis

As gastropods have few real homologous points that could be used in a landmark analysis (Johnston et al., 1991; Stone, 1998), we chose an outline-based technique to capture shell shape. The applied method is the so-called Fast Fourier Transform, which reproduces any given outline (being a set of x,y-coordinates) by fitting in a number of harmonics, i.e. a combination of sine and cosine waves (Davis, 1986; Press et al., 1992; Crampton and Haines, 1996; Haines and Crampton, 2000). The more harmonics one chooses the more accurate the redrawing becomes. For details on the advantages of this technique over other outline-based methods see Haines and Crampton (2000) and Neubauer et al. (2013a). However, this approach ignores size differences and ornamentation features that do not protrude from the outline.

Images of the gastropods were taken highly overexposed to create sharp outlines. As several specimens had slightly damaged apertures, they were photographed from backside. Broken apertural margins were restored with Corel PHOTO-PAINT X4 where necessary. Such minor edits were only performed when there was no doubt about the course of the original shell margin. Additionally, the contrast was enhanced by 100% and the tools “dust and scratches” (parameters: level 80, radius 5) and “noise reduction” (parameters: minimum, percentage 100, radius 1) were applied to reduce outline irregularities that could distort the results of the morphometric analysis (see also Neubauer et al., 2013b). Outlines were captured with TpsDig 2.16 (Rohlf, 2010) using the shell apex as starting point. The resulting x,y-coordinates were processed with the program Hangle, which employs an improved Fast Fourier Transform (Crampton and Haines, 1996; see also Haines and Crampton, 2000). Outlines were smoothed 10 times to reduce potential pixel noise. To reproduce the shell outlines accurately 24 harmonics were chosen (tested with inverse Fourier composition using Hcurve; Crampton and Haines, 1996). Subsequently, curves were matched for starting point, in our case the apex, using Hmatch (Crampton and Haines, 1996). From the variance–covariance matrix of the resulting Fourier coefficients a principal component analysis (PCA) was computed in PAST 2.17c (Hammer et al., 2001). Shell outlines illustrated in the PCA plot were created with the tool “outline trace” (parameters: logo, details moderate, smoothing 25, corner smoothness 0) in CorelDRAW X4. The 3D animated plot was produced from automatized serial imaging in PAST, whereas the 250 single images were set together in a single avi-file with Corel PHOTO-PAINT X4.

### 3.2 Isotope analysis

It has been shown that differences in the isotope signatures of mollusk shells reflect differences in the chemistry of the ambient water and thus can be used as proxies for reconstructing paleo-water conditions (e.g., Grossman and Ku, 1986; Geary et al., 1989; Latal et al., 2004, 2005, 2006; Harzhauser et al., 2007, 2012). In total, the aragonite shells of 30 *Microcolpia* specimens have been analyzed for their δ^18^O and δ^13^C signatures. The shells from Lake Pețea were classified into: the recent shells of *Microcolpia parreyssii parreyssii* (5 specimens, 30 samples), subfossil shells of morphologically typical *M*. *p*. *parreyssii* (7 specimens, 48 samples), subfossil *M*. *parreyssii sikorai* with stepped shells and cylindrical last whorl (7 specimens, 48 samples), and *M*. *p*. *sikorai* (sensu Paucă, 1937) with elongate, keeled or smooth shells (*hazayi*-phenotype sensu Kormos, 1905b; 6 specimens, 50 samples). As outgroup, *Microcolpia daudebartii daudebartii* (Prevost, 1821) shells from the thermal spring Hansybach at Bad Vöslau were measured as well (5 specimens, 22 samples).

Gastropod samples were cleaned in deionized water before analysis. Samples were drilled along the outer spiral of each shell, beginning from approximately 2 mm from the aperture and ending where the whorl became too small to allow the extraction of sufficiently large samples. Only adult shells with fully developed aperture have been selected for isotope analyses. To gather comparable data sets, 3–18 samples were taken along the last whorl (360° from aperture).

Samples for δ^13^C- and δ^18^O-analysis of the shell carbonate were taken from polished slabs with a handheld micro-drill. Sample powders were reacted with 100% phosphoric acid at 70 °C in a Kiel II automated reaction system, and the evolved carbon dioxide gas was analyzed with a Finnigan Delta Plus mass spectrometer at the University of Graz (analytical precision < 0.05‰ for δ^13^C, < 0.1‰ for δ^18^O). The δ^13^C- and δ^18^O-values are corrected according to the NBS19 standard and reported in per mill (‰) relative to the Vienna-PeeDee Belemnite (V-PDB) standard. Temperature estimates are based on the equation of Grossman and Ku (1986) in which the relation between temperature (T), δ^18^O_water_ and δ^18^O_aragonite_ is: T (°C) = 20.6–4.34 (δ^18^O_aragonite_–δ^18^O_water_).

### 3.3 Size, sculpture, and repair marks

Size was measured as shell height and width using a digital caliper. Sculpture was qualitatively recorded, since it is not traceable by the outline analysis. Although some variability is observed, the presence and mode of sculpture could be easily classified into three different states: absent; axial ribs present; 1–2 spiral keel(s) present. The possible states in combination with main outline features and the material available for them are summarized in Table 1.

To test for a potential predator–prey relationship we scored the number of repair marks on fossil and recent shells. A high number of repair marks have been demonstrated to be indicative of selective pressure (e.g., Vermeij and Covich, 1978; West et al., 1991; West and Cohen, 1994; Johannesson, 2001). For the scoring only healed fractures were counted. Broken peristomes were ignored as they might be artificial. Moreover, only healed fractures allow conclusions on the presence of selective pressure in that concern (Vermeij, 1983). Growth cessations parallel to the apertural margin, which occur frequently in gastropods during later ontogeny, were neglected as well. The co-occurrences of repair marks with outline features (coded as presence-absence data) were evaluated using the Dice similarity index to test for significant correlation (Kallio et al., 2011). This index was used as it puts more weight on joint occurrences (Hammer and Harper, 2006).

## 4 Results

**Fig. 3:**
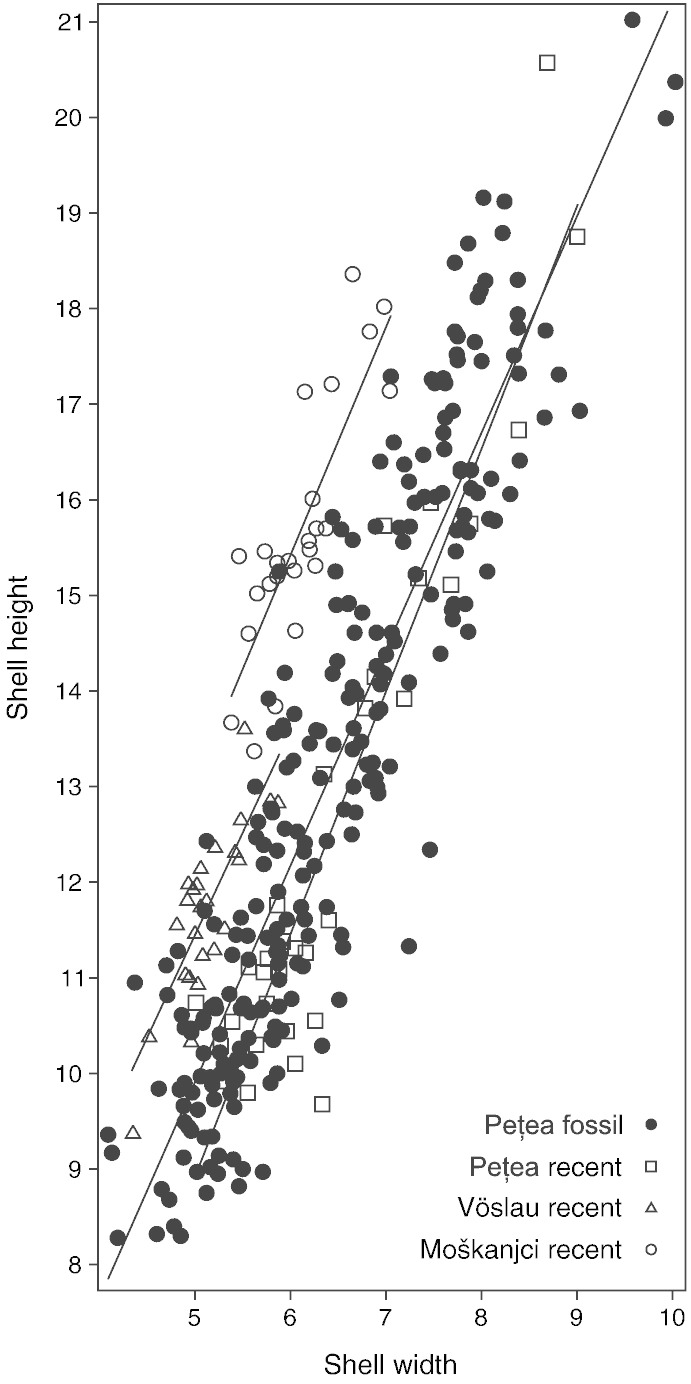
Shell height and width with indication of linear trend lines corresponding to the four groups. Note that *M*. *d*. *acicularis* (Moškanjci) and *M*. *d*. *daudebartii* (Vöslau) are quite well separated, while subfossil and recent Pețea-melanopsids fully overlap.

**Fig. 4:**
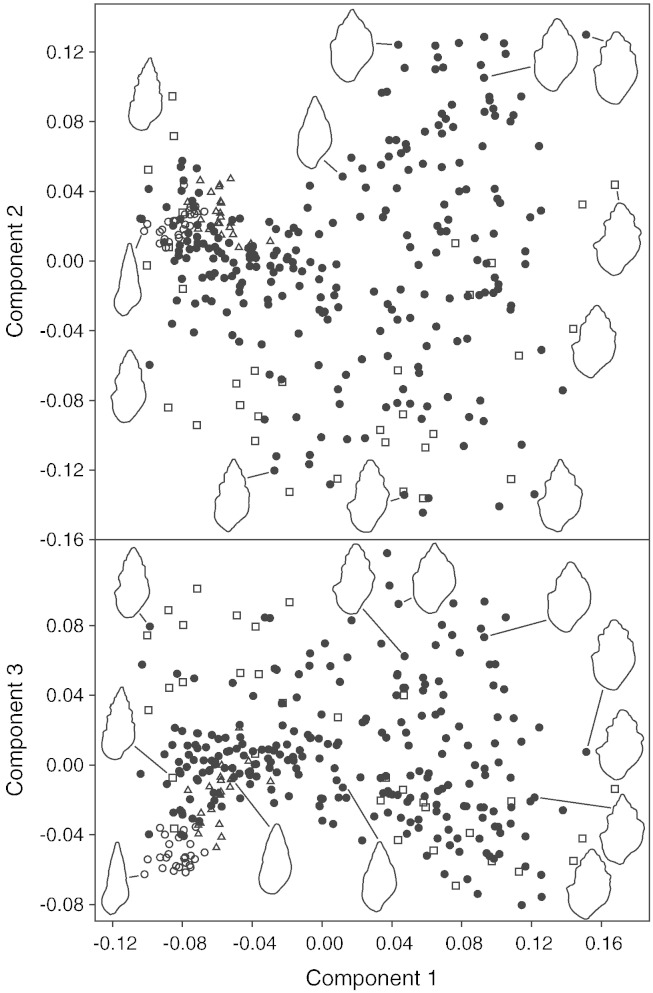
Principal components analysis (PCA) of the Fourier coefficients, reflecting relative morphological distances between specimens. For easier understanding outlines of typical specimens are incorporated (not true to scale). The first three components out of 46 are illustrated, together accounting for 51.6%. Legend: filled circles — subfossil Pețea cluster; squares — *M*. *p*. *parreyssii*, recent; open circles — *M*. *daudebartii acicularis*, recent; triangles — *M*. *daudebartii daudebartii*, recent.

The morphological variability present in the specimens from Lake Pețea is described by three main parameters. First, differences among shell sizes of fully grown individuals (Fig. 3). Second, general shell shape, regarding slender vs. bulky, stepped vs. non-stepped, and high- vs. low-spired forms (Fig. 4). And finally, the presence, mode, and strength of sculpture, being either present as axial ribs or faint to distinct spiral keel(s) or entirely absent.

The PCA plots illustrate the morphological variability of subfossil versus recent specimens (Fig. 4). As indicated by the loading values (not included here), PC 1 (25.613%) is strongly influenced by relative shell width — individuals become more slender from right to left. This coincides with a decreasing degree of whorl stepping. Already PC 2 (17.694%) and PC 3 (8.278%) and especially higher components (PC 4: 6.295%, PC 5: 3.697%) are difficult to interpret and cannot be reliably linked to certain shell parameters; they are produced from a combination of morphological traits. Nevertheless, the analysis shows that subfossil and recent Pețea-melanopsids have a clear morphologically overlap, although both clusters do not have the same morphospace expansion. The smooth and elongated *Microcolpia daudebartii acicularis* remains quite well isolated, while *Microcolpia daudebartii daudebartii* from Bad Vöslau thermal spring shows a considerable overlap with the Pețea-melanopsids. Another very important point is that there is apparently no clustering within the subfossil melanopsids. Thus, the taxonomic concepts and names used up to now for these shells have little support in morphometric analysis. The fact that several recent *Microcolpia parreyssii parreyssii* specimens plot far to the left, near smooth *M*. *d*. *acicularis*, might be a consequence of the limited capability of the analysis to account for small differences in the degree of whorl stepping. These specimens have a near smooth outline and thus appear similar to the elongated *M*. *d*. *acicularis*. To get an impression of the unevenly occupied partial morphospaces of the respective groups we recommend viewing the animated 3D-plot of the principal components analysis (PCs 1–3, same symbols used as in Fig. 4), provided as online supplementary material (XXX).

Shell size is apparently a quite variable factor for both the recent and subfossil melanopsids from Lake Pețea (Fig. 3). Even within the same phenotype the individual size varies between adult specimens (Fig. 2). The Pețea melanopsids are well separated from *Microcolpia daudebartii daudebartii* and *Microcolpia daudebartii acicularis*. Variability of proportions between shell height and width plays a lesser role in all four groups. This is shown by the low lateral spread of the clusters in Fig. 3. Given the nearly parallel trend lines, this factor does not help to distinguish the groups either. For this reason we will not amplify this issue. Likewise, the presence and expression of sculpture are highly variable. Nearly any possible combination of stepped and non-stepped spires with ribbed, keeled or absent sculpture occurs (Table 1). The combinations “stepped and keeled” and “non-stepped and ribbed” are rarely detected. Given our rich collection a sampling bias is rather unlikely. Data for the alteration of sculpture through time is provided by Kormos (1905b) and Sümegi et al. (2012b).

Repair marks were observed on 39 shells in total (14.0%). The evaluation of the co-occurrence of repair marks with distinct features resulted in similar values (Dice similarity measure, stepped outline: 0.229; ribbed: 0.163; keeled: 0.232; non-stepped: 0.206; smooth: 0.221). Ribbed shells seem to have slightly fewer repair marks (albeit this could be biased from the smaller sample size).

### 4.1 Isotope data

**Fig. 5:**
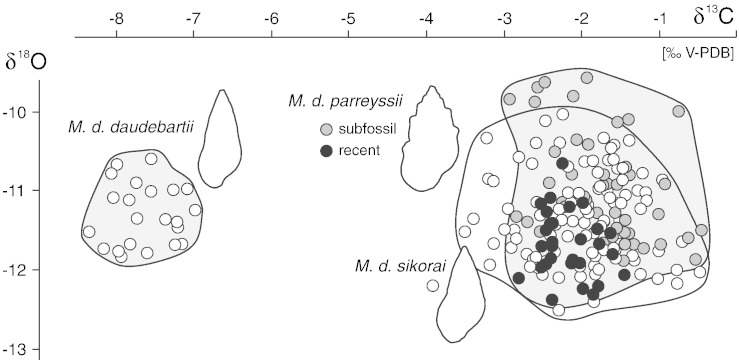
δ^18^O/δ^13^C-cross plot of all data (30 specimens, 198 samples). Note the distinct overlap of subfossil and recent, as well as smooth and sculptured, shells of Lake Pețea melanopsids. They are well separated from the morphologically similar and closely related thermal spring snail *M*. *daudebartii daudebartii* from Austria.

The δ^18^O values range from − 12.37‰ to − 10.65‰ (mean − 11.68‰, σ = 0.41) in *Microcolpia parreyssii parreyssii* from Lake Pețea (recent), from − 11.88‰ to − 9.56‰ (mean − 10.96‰, σ = 0.66) in *M*. *p*. *parreyssii* (subfossil), from − 12.51‰ to − 10.11‰ (mean − 11.25, σ = 0.54) in *Microcolpia parreyssii sikorai* (stepped shells) and from − 12.17‰ to − 10.03‰ (mean − 11.41‰, σ = 0.57) in *M*. *p*. *sikorai* (elongate shells) (Fig. 5).

The δ^13^C values of the shells range from − 2.84‰ to − 1.48‰ (mean − 2.20‰, σ = 0.33) in *Microcolpia parreyssii parreyssii* (recent), from − 2.96‰ to − 0.49‰ (mean − 1.76‰, σ = 0.60) in *M*. *p*. *parreyssii* (subfossil), from − 3.43‰ to − 1.19‰ (mean − 2.00‰, σ = 0.54) in *Microcolpia parreyssii sikorai* (stepped shells) and from − 3.54‰ to − 0.51‰ (mean − 2.04‰, σ = 0.74) in *M*. *p*. *sikorai* (elongate shells).

The stable isotope composition of the recent *Microcolpia daudebartii daudebartii* (Prevost, 1821) from Vöslau displays similar ranges from − 11.83‰ to − 10.59‰ for δ^18^O (mean − 11.29‰, σ = 0.39) but much more negative δ^13^C values ranging from − 8.39‰ to − 7.02‰ (mean − 7.67‰, σ = 0.39). For the complete data table see the supplementary online material.

## 5 Discussion

### 5.1 Environmental reconstruction

In summary, we observe a pattern of morphological evolution or, more precisely, a change of the occupied morphospace during a few thousand years and within an extraordinarily small geographic area (Fig. 1). While the extant assemblage only includes stepped and ribbed shells, the subfossil cluster covers a much greater diversity of shapes, involving smooth, stepped, keeled, and ribbed morphologies in almost every imaginable combination (Fig. 2). The main questions to be tackled are how and why such a considerable morphological shift happened in such a small lake and over such a short interval.

Today Lake Pețea is restricted to an area of several hundred square meters with a maximum depth of 3 m (Covaciu-Marcov et al., 2003). It has an average temperature of 30 °C, with seasonal differences ranging from 26 °C in winter to 35 °C in summer (Telcean and Cupşa, 2013). Historical collections and early papers (e.g., Kormos, 1905b) indicate that the former extent was somewhat larger, but exact information about the lake size during the Pleistocene-Early Holocene is not available. Based on the map and descriptions of Kormos (1905b), an original diameter of a few hundred meters is likely. There is no information about any record of the mentioned morphologies outside the lake, so a bias from immigration is unlikely.

It is difficult to reveal the actual morphospace occupation per time slice as no stratified collections are available to us. Brusina (1903) gives an overview of the occurring morphologies but without a stratigraphic frame. Kormos (1905b) was the only one who gave a section-based succession of certain morphologies through time, together with information about the lithology the shells derive from. This indicated indeed a rather gradual change of smooth to stepped/ribbed phenotypes. Similarly, Sümegi et al. (2012b) referred to a core when giving morphological characteristics of occurring melanopsids and radiocarbon datings of certain limestone layers. A synthesis of the data from both studies allowed an approximation of the temporal succession combined with lithological information (Fig. 1). After Sümegi et al. (2012b) the oldest sediments formed around 15,000–13,000 years BP. During the early lake stages (latest Pleistocene, up to 11,600 years BP) only smooth, elongate, non-stepped morphologies are present in the limestone-dominated sediments (*hazayi*-phenotype). With the beginning of the Holocene epoch stepped (*sikorai*) and keeled (*mucronifera*, *staubi*) phenotypes co-occur with the elongate ones for a short period. In the following layer only these stepped and keeled morphologies can be found; elongate, non-stepped forms are not present anymore. This coincides with the deposition of peaty clay and the co-occurrence of a rich pulmonate-dominated mollusk fauna. The subsequent interval displays a short return to carbonate sedimentation with a low diverse mollusk fauna. Kormos (1905b) explicitly states that this interval shows the transition from the smooth and stepped to ribbed phenotypes (*sublanceolata*). Although the latter does not occur before the following layer, there are several shells found with first intentions of ribs. The interval above is probably the most interesting as it contains the first ribbed morphologies. It consists of a single 45 cm-thick bed of peaty clay. In its lower part smooth phenotypes still coexist with the ribbed forms (around 8500–8400 years BP), whereas they become fully displaced in the upper part. Above follow again marly sediments bearing only ribbed and stepped shells; depending on the degree of stepping they are variably termed in the literature as *sublanceolata*, *hungarica* or *parreyssii* (from ca. 4000 years BP to present).

A more detailed insight into past environmental conditions and variability is provided by the isotope data. Major changes in hydrology, triggering the development or disappearance of certain shell characters, might be expected to be at least partly reflected in the stable isotope signatures. Although Shanahan et al. (2005) documented isotope variability in shell aragonite of freshwater gastropods under constant temperature and δ^18^O_water_ conditions that was larger than predicted by equilibrium models, the isotope signatures are still a valuable tool to reconstruct environmental shifts (Dettman et al., 2003; Leng and Marshall, 2004).

The selection of shells analyzed comprises a broad range of morphologies, representing all major phenotypes described from Lake Pețea. The δ^18^O/δ^13^C cross plot (Fig. 5) clearly shows a near complete overlap of the isotope signatures of the specimens from Lake Pețea. There is weak difference between the recent and subfossil shells and there is no separation between elongate-smooth and stepped-ribbed shells. Sümegi et al. (2012a) suppose that fluvial influx did not influence the water chemistry of the lake after its very initial phase. Therefore, the δ^18^O values of the gastropods are expected to have been influenced only by water temperature and the δ^18^O_spring-water_ and δ^18^O_precipitation_. No significant negative δ^18^O excursions linked to major influx by isotopic light precipitation are captured in the isotopic records. Therefore, we interpret the lowest δ^18^O to reflect the warmest temperatures of ambient lake water. There is no information on Holocene and Pleistocene δ^18^O_lake-water_ available, but using the recent water temperature (oscillating around 30 °C ± ~ 5 °C) for the recent shells results in a δ^18^O_lake-water_ of − 9 to − 10‰ (using the equation of Grossman and Ku, 1986). Accepting this δ^18^O_water_ for the subfossil shells results in similar mean temperatures for all subfossil shells (*Microcolpia parreyssii parreyssii*: 26.9 °C, σ = 2.9; *Microcolpia parreyssii sikorai*, stepped shells: 28.2 °C, σ = 2.4; *M*. *p*. *sikorai*, elongate shells: 28.9 °C, σ = 2.5). A separation of these phenotypes based on calculated ambient water temperatures is impossible. Of course these calculations are only rough estimates as we cannot exclude that the δ^18^O_lake-water_ had changed during the latest Pleistocene and Holocene. Nevertheless, it is a realistic assumption that the stable isotope composition of the ambient water did not change markedly during the life-span of an individual gastropod. Therefore, the data may be used as estimates of seasonal temperature range in the lake water (Harzhauser et al., 2012). This range is rather low in the thermal lake, attaining 5–10 °C (Craciun, 1997; Mintaş et al., 2012) and is also reflected by the recent specimens of *M*. *p*. *parreyssii* (7.2 °C, no. 27). As only the last whorl was analyzed in our study, only a part of the full seasonal temperature range will be captured. Nevertheless, the documented variability is comparable to that of the recent specimens: 5.66 °C in *M*. *p*. *parreyssii* (no. 7) and 7.48 °C in stepped *M*. *p*. *sikorai* (no. 14). These data indicate that no dramatic change in seasonality of water temperature occurred. Hence, this factor does not explain the observed shift in morphospace occupation.

**Fig. 6:**
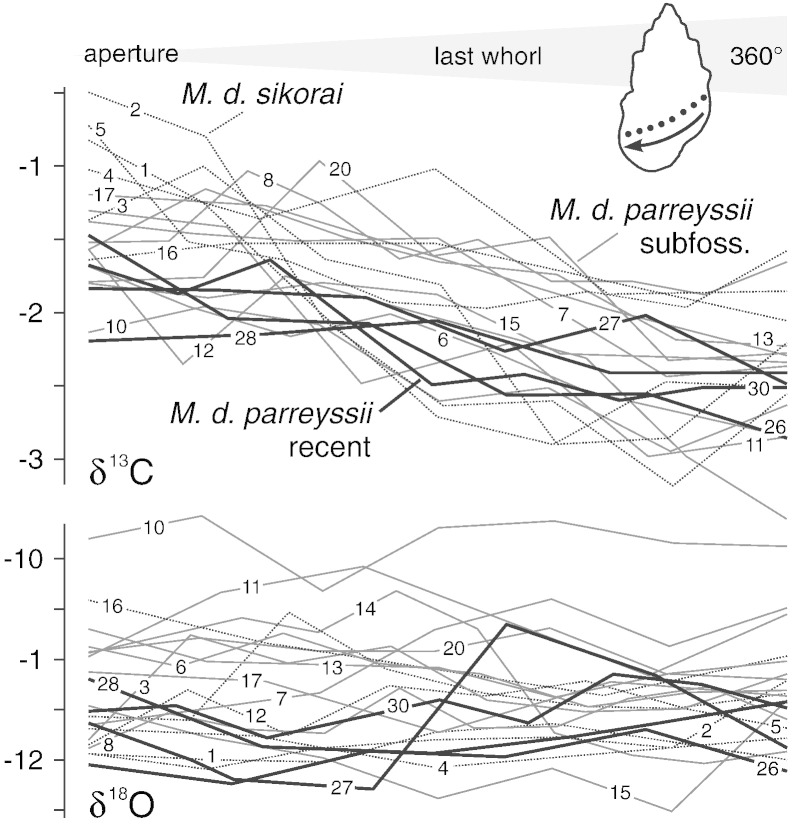
Sclerochronology of representative phenotypes of the recent and subfossil *M*. *p*. *parreyssii* (Philippi, 1847) and subfossil *M*. *p*. *sikorai* (Brusina, 1903) from Lake Pețea, Romania. Ontogenetically youngest samples, taken close to the aperture, appear to the left. The numbers next to the lines correspond to the specimen numbers as given in STab. 1 (supplementary online material). While the overall stable δ^18^O values suggest fairly constant water temperatures in the thermal pond, the clear trend in δ^13^C values indicates considerable seasonal shifts in the carbon pool.

The δ^13^C values of all subfossil *Microcolpia* shells from Lake Pețea have very similar ranges and do not allow any separation. The recent shells display a narrower range but cluster close to the mean of the subfossil data set (the narrower range may be due to the smaller sample number). The observed ranges are characterized by a distinct trend from depleted toward heavier δ^13^C values during ontogeny in the recent and subfossil specimens (Fig. 6). This trend suggests that the last whorl developed over about half a year. Specimens 1, 5, 2, and 3 show also the reversed trend toward heavier values in earlier stages of ontogeny as expected if the pattern was caused by seasonality. δ^13^C_shell_ of mollusks is considered to be mainly influenced by dissolved inorganic carbon (DIC) of ambient water (McConnaughey and Gillikin, 2008). Nevertheless, influence by dietary carbon and metabolic processes is suggested in many studies to contribute to animals' carbon budget as well (Dettman, 1999; Gajurel et al., 2006). Lorrain et al. (2004), Gillikin et al. (2007), and Harzhauser et al. (2011) argued that the contribution of metabolic carbon in mollusks increases with size and age, leading to a downshift trend in δ^13^C_shell_ especially in late stages of growth. As the *Microcolpia* shells display an opposite trend toward heavier values, this scenario is unlikely and the change is most probably related solely to changing DIC values. There is little reason to assume that thermal spring water DIC displays significant seasonal variation (Shanahan et al., 2005). Therefore, the observed isotopic shifts are forced by photosynthetic productivity of plants and algae, preferentially incorporating light ^12^C and enriching the lake water with ^13^C (Leng and Marshall, 2004). Especially in a very shallow and small setting such as Lake Pețea, supplied by spring water with constant δ^13^C_water_, the DIC pool will be strongly modified by vegetation periods (Leng and Marshall, 2004). As the positive shift in δ^13^C_shell_ is documented for the recent and subfossil shells alike, the seasonal oscillation of DIC was clearly not responsible for the shift in morphospace occupation.

Shells explicitly deriving from the peaty deposits are not present, so we cannot quantify the actual impact of this period on the melanopsids. However, as both oxygen and carbon isotopes are obviously constant throughout the rest of the succession, below and above the peaty layer, morphologies are obviously not directly linked to the trophic state.

The ancestral smooth phenotype found during the latest Pleistocene and early Holocene of Lake Pețea is reminiscent of the closely related extant *Microcolpia daudebartii daudebartii* (Prevost, 1821). This could point to comparable ecological conditions, different from those favoring the development of the ribbed and shouldered morphologies. This species lives in a small thermal spring at Vöslau (Austria, Vienna Basin) with constant water temperature of 22–24 °C, a δ^18^O-value of − 11.00‰ and a δ^13^C-value of 9.55‰ (Zötl and Goldbrunner, 1993). Using this δ^18^O_water_ in the equation of Grossman and Ku (1986), a mean temperature of 21.9 °C (σ = 1.7) for the ambient water during shell growth is given for the herein analyzed specimens. The individual temperature ranges of the specimens vary from 1.16 °C to 3.21 °C, suggesting very weak seasonality effects. The total temperature range of 5.38 °C with a lowest value of 18.80 °C may be influenced by shells of animals that lived some meters downstream within the brook.

Despite the similarities of oxygen signatures of *Microcolpia daudebartii daudebartii* and those of Lake Pețea shells, the carbon signatures are significantly more negative and lack the trend toward heavier values observed in Lake Pețea. As for ribbed and shouldered specimens, smooth and more or less elongate shell morphology is not linked to a certain isotope regime. Both phenotypes have developed under near identical stable isotope values of the ambient water, whereas the same elongate smooth phenotype may emerge under strongly deviating conditions. This indicates that neither temperature nor nutrition (as reflected in DIC composition) are the key-factors influencing morphospace occupation in *Microcolpia*.

### 5.2 Morphological evolution

The synthesis of old and new data indicates a two-stage evolutionary event for the Lake Pețea melanopsids. The first stage involves the phenotypic evolution from entirely smooth and elongate to stepped and, alternatively, keeled shells around the Pleistocene-Holocene boundary (Fig. 1). The second comprises the shift from these stepped and keeled shells to the ribbed morphologies. Both episodes seem well separated as smooth-elongate and ribbed morphologies are never recorded for the same bed. However, the linkage of the two shifts with changes in the environment is only evident for the second interval, where the occurrence of ribbed morphologies is coupled with the deposition of peaty clay.

Morphological change in melanopsid gastropods was often discussed to be an expression of extrinsic factors (e.g., Glaubrecht, 1993, 1996; Michel, 1994; Bandel, 2000; Heller and Sivan, 2002). Elkarmi and Ismail (2006) observed for populations in the river Jordan that smooth types occur in springs and oasis, while ribbed morphologies are typical for streams and rivers. A comparable relation of phenotypic plasticity with hydrological conditions was also documented for fluvial pleurocerid gastropods in Arkansas, USA (Minton et al., 2011). Species tend to be thinner, smoother, and less inflated in headwater areas, while they become thicker, more sculptured, and more inflated in downstream direction — a relation known as Ortmann's law (Ortmann, 1920). Varied hydrodynamic conditions were also discussed as potential agent for morphological evolution in Late Miocene Lake Pannon melanopsids (Geary et al., 2002; Neubauer et al., 2013a). Still, a strong impact from changing water energy can be ruled out for Lake Pețea as fluvial input is limited (Covaciu-Marcov et al., 2003; Sümegi et al., 2012a). Moreover, fluvial-derived waters with usually lighter oxygen values would be detectable in the isotope analyses.

Another frequently cited trigger for the development of sculpture is predator–prey coevolution (e.g., Vermeij and Covich, 1978; Vermeij, 1983; West et al., 1991; Martens et al., 1994; West and Cohen, 1994; Johannesson, 2001). For Lake Pețea such a scenario was even demonstrated to be the reason for the color pattern evolution of a fish species *Poecilia sphenops* (Valenciennes in Cuvier and Valenciennes, 1846). Black phenotypes in combination with the shade-providing endemic water lily *Nymphaea lotus thermalis* (De Candolle) Tuzson, 1907 are hidden more effectively from other predatory fishes than their lighter colored counterparts resulting in higher fitness (Petrescu-Mag et al., 2008). For our material repair marks were observed on only 39 shells in total (14.0%). The low and throughout similar numbers of co-occurrences between repair marks and distinct morphological features suggest that the evolution of sculpture was not induced by a predator–prey relationship. In such a case ornamented shells would be expected to have significantly more repair marks as the snails survive predation more often than non-sculptured ones. Furthermore, although the extant phenotypes are highly ribbed and stepped and thus obtain a higher constructional stability than smooth and elongate ones, the throughout very thin, fragile outer lip remains a blind spot that could be easily crushed by potential predators.

### 5.3 Bottleneck and genetic drift

All these negative evidences imply that there is no direct interdependence between morphology and environment. In evolutionary studies a factor readily underestimated and often neglected is genetic drift. It is often disregarded when a pattern looks convincingly directional, as it seems for the present case. However, it was repeatedly discussed and modeled that drift has an important influence on evolution. It allows a population to move against natural selection, what may open new evolutionary pathways by approaching different adaptive optima or zones (e.g., Lande, 1976; Raup, 1977; Ackermann and Cheverud, 2004; Estes and Arnold, 2007). This holds particularly true for small populations (Raup, 1977). For these a well-known phenomenon is the “bottleneck effect”. The idea behind this theory is based on shifting population size. A once big population becomes reduced, no matter for what reason, and re-expands after the crisis. The intermittently small population may suffer from a loss and/or shift of allele frequencies, i.e. genetic variability (Maruyama and Fuerst, 1985; Amos and Hoffman, 2009). Given time and the influence of drift, this might lead to completely varied allele dominances occasionally linked with changed phenotypic expressions in the newly rising population. A famous example is the human evolution where this effect has recurrently led to genetic revolutions (e.g., Ambrose, 1998; Hawks et al., 2000; Amos and Hoffman, 2009).

Exactly this theory might be applicable in our case. The accumulation of peaty clay evidences the high input of organic matter (e.g., Treese and Wilkinson, 1982; Opluštil and Vízdal, 1995). At that time Lake Pețea was a very shallow, swampy, highly eutrophic, densely vegetated environment. Although the high input of organic matter generally increases resources for the detrital-feeding gastropods (e.g., Pieczyńska et al., 1999; Timm et al., 2006), melanopsids are readily marginalized by species better adapted to eutrophic environments. This involves pulmonate snails such as lymnaeids and planorbids (e.g., Fechter and Falkner, 1990; Glöer, 2002). During both intervals of increased peat deposition Kormos (1905b) and Sümegi et al. (2012a) noted a rise in gastropod diversity paralleling the first appearance of ribbed melanopsids. Particularly planorbids increase in number of individuals and species (5–6 in peaty clays compared to maximally 2 below, in between, and directly above the peaty layers; Kormos, 1905b). Moreover, population size might have strongly decreased. A study on the impact by eutrophication on invertebrate communities in several Polish lakes showed that snails can constitute the most abundant group in low trophic systems (with up to 50% of all macroinvertebrates), while they can decline down to a few percent in eutrophic counterparts (Pieczyńska et al., 1999). A similar result was presented by Deng et al. (2005) for a Chinese lake, where density and biomass of certain bivalve and gastropod species were reduced during progressive eutrophication; in hypertrophic stages the mollusks were completely absent. Additionally, the decay of plant matter limits the available habitat (Pieczyńska et al., 1999).

Concluding, it is very likely that the establishment of the eutrophic, swampy conditions diminished the melanopsids' population size drastically. This bottleneck promoted the action of genetic drift, which resulted in the seemingly accidental evolution of stepped and ribbed morphologies. The fact that shells with the first intentions of ribs occurred in the carbonate interval between the peaty deposits is not surprising at all as genetic drift and its phenotypic expression take some time (Lande, 1985). Consequently, the increasing influence of genetic drift may have started already in the first peaty interval and persisted up to the second phase. Since the morphological spectrum did not change significantly afterwards, the influence of genetic drift might have been limited by then as well.

The first event in turn, with the evolution of stepped and keeled shells, could not be reliably linked to environmental change. Therefore, we interpret it as phenotypic evolution apparently without (or at least minor influence of) natural selection. This claim is supported by the absence of selective agents, be it by predator–prey coevolution, intraspecific competition for resources, hydrodynamic conditions, substrate properties, or water chemistry — all of which could be reliably excluded.

### 5.4 What if?

Although we could exclude common selective agents, we cannot exclude the possibility that natural selection still triggered or at least influenced the observed pattern and we simply lack the resolution or the means to point it out. Given the restricted temporal frame, a real speciation event can be largely excluded — be it because of natural selection, genetic drift, or a combination of both. Common species divergence times exceed the present time frame by several magnitudes, even for the more rapid case of sympatric evolution (following Bookstein, 1987; Futuyma, 2009; Hunt, 2012; Rabosky et al., 2013). Still we might face the initial phase of speciation, or in other words, genotypic and subsequent phenotypic differentiation and divergence because of reproductive isolation. This might arise from attraction to different adaptive optima (e.g., Simpson, 1953; Lande, 1976, 1986; Estes and Arnold, 2007; McGhee, 2007; Gavrilets, 2010) and subsequent selection for varied ecological or behavioral aspects, but requires some kind of geographical and/or behavioral barrier (e.g., Futuyma, 2009; Sadedin et al., 2009). In such a small environment like Lake Pețea, with several hundred to a few thousand square meters during its former extent, it is certainly difficult to achieve isolation. As mentioned above, there is no sign of habitat differentiation, neither in the past nor in the present lake, and immigration events are highly improbable as no findings are documented from outside the lake (Kormos, 1905b; Covaciu-Marcov et al., 2003). The establishing swampy conditions in the second interval of morphological evolution affected the whole lake and restricted and changed rather than diversified the habitat.

The small, uniform habitat would if at all rather argue for sympatric evolution. For this still debated mode of evolution several theoretic scenarios have been established (for reviews see Coyne and Orr, 2004; Fitzpatrick et al., 2008, 2009; Hendry, 2009). Sympatric evolution may occur as a consequence of abruptly occurring environmental shifts resulting in rapidly diverging adaptive optima (Estes and Arnold, 2007; Futuyma, 2009). Individuals then trace the new optima, resulting in a genotypic (and occasionally phenotypic) divergence. In such a scenario intermediate genotypes usually serving as gene transmitter between the two evolving species have a lower selective advantage and are rapidly suppressed. The great variety of intermediate morphologies present in Lake Pețea makes this idea quite unlikely. Another theory favors sympatric speciation along an environmental gradient. Intrinsic adaptive processes in a population might produce sharp geographical differentiation even in the absence of abrupt environmental changes (Doebeli and Dieckmann, 2003). In this model particularly the ecological contact between individuals or subpopulations is a requirement for local disruptiveness to happen (e.g., over assortative mating). Again, there is no environmental gradient identifiable in the thermal lake system. In particular no vertical or horizontal habitat differentiation is present (Covaciu-Marcov et al., 2003). A third hypothesis states that sympatric evolution can be prompted by competition for resources (Dieckmann and Doebeli, 1999). This is precluded by the perfectly matching δ^13^C-isotope signatures; the overlap for the recent and subfossil assemblages evidences equivalent dietary preferences.

Consequently, it was not possible to detect any scenario in which evolution by selection is the favorable hypothesis. Moreover, the evolutionary pattern appears one-directional, with a step-wise shift of the occupied morphospace in the same lineage, rather than a split into two or more lineages. Still we cannot rule out the influence of natural selection. A favorable approach to substantiate and refine the present interpretation would be to incorporate more specimens from stratified collections and to perform more detailed analyses of isotopic compositions and properties of the sediment.

### 5.5 A Miocene near-counterpart

The evolutionary pattern reminds of the late Middle Miocene Lake Steinheim in southern Germany. This small crater lake witnessed an incredible endemic evolution of planorbid snails, with 17 species deriving from a single ancestor (e.g., Hilgendorf, 1867; Mensink, 1984; Reif, 1985; Gorthner, 1992; Nützel and Bandel, 1993; Rasser, 2013a, b). The trigger for evolution is still debated. According to Nützel and Bandel (1993) environmental pressure promoted phenotypic diversification, but without any functional adaptation. Gorthner (1992) suggested a barrier formed by *Chara* belts to allow microgeographical isolation, similar to extant Lake Ohrid (Albrecht and Wilke, 2008; Hauffe et al., 2011; Schreiber et al., 2012). The similarities with Lake Pețea are, however, only superficial. Lake Steinheim was larger (ca. 3.5 km in diameter), the endemic evolution persisted over a much longer time interval of several hundred thousand years, and the environmental setting as crater lake with increased salinity differs markedly from the Romanian situation (Rasser, 2013b). The influence of thermal water on planorbid evolution considered by earlier workers (Gottschick, 1920; Wenz, 1922) was later rejected by Bajor (1965) based on isotopic analysis. Besides, there is no comparable evolutionary pattern in lacustrine systems known to us.

An influence from the global climate change at the Pleistocene–Holocene boundary can be excluded. Although Greenland isotope and pollen records indicate a strong rise of annual mean temperature beginning at the boundary and persisting up to ca. 7800 years BP (e.g., Davis et al., 2003; Nesje et al., 2004), the rather isolated temperature regime in the thermal spring and the constant δ^18^O-isotope values rule out this large-scale impact.

### 5.6 Taxonomic implications

The implications of the present study on the taxonomy of the *Microcolpia parreyssii*-group are summarized here. The analysis showed that most of the names applied to the variable morphologies are not tenable. Morphologies pass into each other, making most boundaries between taxonomic units arbitrary. Moreover, as outlined above the syntopic evolution of distinct species is unlikely. The most reliable morphological separation is guaranteed when incorporating the factor time. Since there is still considerable variability within the single time slices, the most practicable taxonomic separation is to distinguish ribbed and non-ribbed forms. We do not separate stepped vs. non-stepped morphologies as this characteristic is highly variable within all time-slices, to a lesser extent even for the recent populations. We follow Paucă (1937) who acted as First Reviser and synonymized the numerous taxa of this group and chose “*Melanopsis sikorai* Brusina, 1903” to be the accepted name. As we rule out a speciation event we consider *M*. *sikorai* as a subspecies of *M*. *parreyssii*. Consequently, the only two accepted names are *Microcolpia parreyssii parreyssii* (Philippi, 1847) and *Microcolpia parreyssii sikorai* (Brusina, 1903). Detailed synonymy lists for the involved species-group names are provided in the Appendix.

Finally, both the PCA and the measurements show that the two recent subspecies *Microcolpia daudebartii acicularis* and *Microcolpia daudebartii daudebartii* can be separated sufficiently. Although they plot close together, the two clusters do not overlap. This is best visible in the 3D plot (online supplementary material). This result confirms the established taxonomic separation on subspecies level used by most authors (e.g., Fischer and Müller, 1996; Fischer, 2002; Glöer, 2002; Glöer and Sîrbu, 2005; De Jong, 2012).

## 6 Conclusion

The synthesis of old and new data gives a quite detailed picture of the evolutionary mechanisms in Lake Pețea *Microcolpia* during the Holocene. The expanding and successively shifting morphospace, visible as a trend toward stepped and sculptured shells was probably initiated by an environmental crisis. Massive organic input into the small thermal lake limited available habitats and boosted eutrophication. The increasing diversity and individual abundance of pulmonate snails together with the stressful ecological conditions probably marginalized the melanopsids and reduced population size. Data from the previous studies and herein presented isotopic analyses make the influence of natural selection unlikely. Given the time interval of few thousand years, a speciation event can be ruled out as well. The most probable hypothesis involves a bottleneck effect as a result of the environmentally induced population decline, favoring the action of genetic drift. This produced a genotypic and the visible phenotypic shift toward stepped and ribbed shells.

Particularly in paleontological studies directionally-seeming events are often linked with speciation. The present study emphasizes that even an “obvious trend” has to be carefully and objectively investigated in order to detect the underlying mode of evolution — if present at all.

The following is the supplementary data related to this article.

Supplementary Table 1 Values of isotopic analyses for studied Microcolpia shells.

Supplementary video Animated 3D-plot of the principal component analysis (PCs 1–3). Symbols are equal to those used in Fig. 4.

Supplementary data to this article can be found online at http://dx.doi.org/10.1016/j.palaeo.2014.08.015.

## Figures and Tables

**Table 1:** Available material for the present study, grouped into morphological states. Each percentage value corresponds to the relative abundance of a morphological state compared to the total number of subfossil *Microcolpia*. Former identifications of the different morphologies are included as well.

Taxon/morphology	Age	Number	Percentages	Locality	Collection	Other/former names
*Microcolpia parreyssii parreyssii* (Philippi, 1847)	Recent	32		Băile 1 Mai, Romania	NHMW Moll.	
*Microcolpia daudebartii daudebartii* (Prevost, 1821)	Recent	25		Bad Vöslau, Austria	NHMW Moll.	
*Microcolpia daudebartii acicularis* (Férussac, 1823)	Recent	25		Moškanjci, Slovenia	NHMW Moll.	
Stepped + ribbed *Microcolpia*	Subfossil	71	28.98	Băile 1 Mai, Romania	NHMW Geol.	parreyssii, themaki, hungarica
Stepped + keeled *Microcolpia*	Subfossil	10	4.08	Băile 1 Mai, Romania	NHMW Geol.	
Stepped + smooth *Microcolpia*	Subfossil	48	19.59	Băile 1 Mai, Romania	NHMW Geol.	sikorai
Non-stepped + ribbed *Microcolpia*	Subfossil	3	1.22	Băile 1 Mai, Romania	NHMW Geol.	sublanceolata, szontaghi
Non-stepped + keeled *Microcolpia*	Subfossil	46	18.78	Băile 1 Mai, Romania	NHMW Geol.	mucronifera, tothi, hazayi, staubi
Non-stepped + smooth *Microcolpia*	Subfossil	67	27.35	Băile 1 Mai, Romania	NHMW Geol.	franciscae, vidovici, hazayi

## Appendix A Taxonomic and nomenclatural details

Here we provide synonymy lists for the two accepted taxa of Lake Pețea melanopsids. To keep the lists assessable we only list first descriptions/mentions of the respective names and synonyms. Additionally, nomenclatural remarks on *Microcolpia daudebartii* are provided clarifying its complex history.

Family Melanopsidae H. and A. Adams, 1854

*Microcolpia* Bourguignat, 1884

Type species: *Melanopsis acicularis* Férussac, 1823. Recent, Europe. Subsequent designation by Cossmann (1909) [misspelt as “*Microcalpia*”].

*Microcolpia parreyssii parreyssii* (Philippi, 1847)

Figs. 2A–C, F.

1847 *Melania* (s. *Melanopsis*) *Parreyssii* “v. Mühlfeld” Philippi: 176, pl. 4, fig. 15.

1874 [*Melanopsis parreyssii*] Varietät *β* (*scalaris* Parr.) Brot: 431, pl. 46, fig. 15.

1874 [*Melanopsis parreyssii*] Varietät γ Brot: 431, pl. 46, fig. 14.

1886 [*Melanopsis parreyssi*] *innodata* Westerlund: 123.

1887 [*Melanopsis parreyssii*] var. *glabrata* Clessin: 690, fig. 475.

1904 *Melanopsis hungarica* Kormos: 107, text-fig. 2.

1905b *Melanopsis szontaghi* Kormos: 392, 439, pl. 2, fig. 2.

1905b *Melanopsis sublanceolata* Kormos: 393, 440, pl. 2, fig. 9.

Remarks: This taxon comprises all the ribbed morphologies. Several names were introduced for the varied degrees of the more or less stepped outline, all of which are considered synonymous. The first records of morphologically typical *Microcolpia parreyssii* from Lake Pețea are dated to 3000–4000 years BP by Sümegi et al. (2012a). “*Melanopsis sublanceolata* Kormos, 1905” and “*Melanopsis hungarica* Kormos, 1904” appear already at 8500–8400 years BP (Sümegi et al., 2012a) (Fig. 1).

Philippi (1847) referred to a collection name given by Von Mühlfeld (“in schedis”). Therefore, the authorship has to be passed to Philippi (1847). The original spelling of the species name was “*Parreyssii*”. The use of “*parreysi*” by many subsequent authors is an unjustified emendation (ICZN Article 33.4).

*Microcolpia parreyssii sikorai* (Brusina, 1903)

Figs. 2E, G–H, J.

1903 *Melanopsis sikorai* Brusina: 111.

1903 [*Melanopsis sikorai*] var. *siminina* Brusina: 112.

1903 [*Melanopsis sikorai* var.] *unifilosa* Brusina: 112.

1903 [*Melanopsis sikorai* var.] *bifilosa* Brusina: 112.

1903 [*Melanopsis sikorai* var.] *megaotyla* Brusina: 112.

1903 *Melanopsis themaki* Brusina: 110.

1903 [*Melanopsis themaki* var.] *plicatula* Brusina: 110.

1903 [*Melanopsis themaki* var.] *unifilosa* Brusina: 110.

1903 [*Melanopsis themaki* var.] *bifilosa* Brusina: 110.

1903 [*Melanopsis themaki* var.] *trifilosa* Brusina: 110.

1903 [*Melanopsis themaki* var.] *unicarinata* Brusina: 111.

1903 [*Melanopsis themaki* var.] *megalostoma* Brusina: 111.

1903 *Melanopsis hazayi* Brusina: 112.

1903 [*Melanopsis hazayi*] var. *contracta* Brusina: 112.

1903 [*Melanopsis hazayi*] var. *elongata* Brusina: 112 (non *Melanopsis elongata* Gassies, 1874).

1903 [*Melanopsis hazayi*] var. *megatyla* Brusina: 112.

1903 [*Melanopsis hazayi*] var. *unifilosa* Brusina: 113.

1903 [*Melanopsis hazayi*] var. *bifilosa* Brusina: 112.

1903 [*Melanopsis hazayi*] var. *carinata* Brusina: 112 (non *Melanopsis carinata* Gassies, 1863).

1903 *Melanopsis franciscae* Brusina: 113.

1903 *Melanopsis vidovići* Brusina: 113.

1903 [*Melanopsis vidovići*] var. *plicatula* Brusina: 113 (non *Melanopsis plicatula* Handmann, 1887).

1903 [*Melanopsis vidovići* var.] *tenuis* Brusina: 114.

1903 *Melanopsis tóthi* Brusina: 114.

1903 [*Melanopsis tóthi* var.] *rugosula* Brusina: 114.

1903 [*Melanopsis tóthi*] var. *unifilosa* Brusina: 114.

1903 [*Melanopsis tóthi*] var. *bifilosa* Brusina: 114.

1903 [*Melanopsis tóthi*] var. *trifilosa* Brusina: 114.

1903 [*Melanopsis tóthi*] var. *quadrifilosa* Brusina: 114.

1903 [*Melanopsis tóthi*] var. *multifilosa* Brusina: 114.

1903 [*Melanopsis tóthi*] var. *unicingulata* Brusina: 114.

1903 [*Melanopsis tóthi*] var. *bicingulata* Brusina: 115.

1903 *Melanopsis staubi* Brusina: 115.

1903 [*Melanopsis staubi* var.] *carinata* Brusina: 115 (non *Melanopsis carinata* Gassies, 1863).

1903 [*Melanopsis staubi*] var. *costulata* Brusina: 115.

1905b *Melanopsis mucronifera* Kormos: 395, 442, pl. 2, fig. 1.

Remarks: Many names were introduced for this particularly variable taxon, comprising all the non-ribbed shells. This includes the slender-elongate forms, the stepped, and the keeled ones. Since all these phenotypes exhibit considerable variability and partly merge into each other, no reliable separation is feasible. The oldest occurrences of taxa which are considered to be synonyms of *Microcolpia parreyssii sikorai*, have been dated by Sümegi et al. (2012a) at 15,000–13,000 years BP (“*Melanopsis hazayi* Brusina, 1903”) and 11,600 years BP (“*Melanopsis sikorai*”, “*M*. *mucronifera* Kormos, 1905”, “*Melanopsis staubi* Brusina, 1903”).

*Microcolpia daudebartii* (Prevost, 1821)

Fig. 2D.

1821 *Melanopsis daudebartii* [Prevost]: 136.

1823 *Melanopsis audebartii* Prevost — Férussac: 160.

1824 *Melanopsis audebardi* — Prevost: 264.

Remarks: *Microcolpia daudebartii* has its type locality at Baden in Austria and is now endemic to the thermal springs at Bad Vöslau and Bad Fischau (Austria) (Fischer, 2002). There is considerable confusion about the correct name and its spelling of this taxon in the literature. It was first mentioned in the year 1821 as “*Melanopsis Daudebartii*” on page 136 in an article in the Bulletin des Sciences, par la Société Philomathique de Paris with the title: “Sur une nouvelle espèce de mollusque testacé du genre Mélanopside, par M. Constant-Prevost. (Extrait)”. The locality of the species is indicated as thermal spring at Baden in Austria. In subsequent papers, Prevost was considered to be the author of this article. From the title and text, however, it is obvious that the article is an “Extrait” of a talk given by Prevost earlier, summarized by an anonymous author. Following ICZN (1999) Article 50.2 and Recommendation 51D the correct citation is *M*. *daudebartii* [Prevost] 1821.

The presentation conducted in or before 1821 was published by Prevost (1824: 264). In this paper he gave a detailed description, but used a different spelling, i.e. “*Melanopsis audebardi*”. He described the type locality as artificially enlarged basin, which acts as reservoir of a thermal spring with sulfur water at Baden in Austria. On the frontispiece Prevost (1824) indicated that the content was presented already in November 1822. Unfortunately, Férussac (1823: 160) added to the confusion in his monograph on *Melanopsis* by using the name “*M*. *audebartii* Prevost”. He denoted the thermal spring at Weslau (= Vöslau) as locality and indicated that the material came from C. Prevost and Partsch. Férussac (1823) added a very short Latin diagnosis – “*minor*, *unicolor*” – to state the difference from his newly described *Melanopsis acicularis*. The confusing situation even deduced Bourguignat (1884) to treat “*M*. *audebartii* Férussac, 1823” as *Microcolpia* and “*M*. *audebardi* Prevost, 1824” as *Fagotia*.

Neither Férussac (1823) nor Prevost (1824) referred to the original spelling “*daudebartii*”. However, an emendation of the original spelling would be based on weak ground as all three variants (*daudebartii*, *audebartii*, *audebardi*) can be based on spellings used by Férussac himself (e.g., d'Audebard de Férussac, 1812; d'Audebard de Férussac, 1814; d'Audebard de Férussac, 1823). Concluding, there is little doubt that all three papers (Prevost, 1821; Férussac, 1823; Prevost, 1824) referred to the very same taxon.
