# Supplementary figures and images for: Population bottleneck triggering millennial-scale morphospace shifts in endemic thermal-spring melanopsids
Source: Palaeogeogr Palaeoclimatol Palaeoecol. 2014 Nov 15;414:116–28. doi: 10.1016/j.palaeo.2014.08.015 (PMC4375792; doi:10.1016/j.palaeo.2014.08.015)

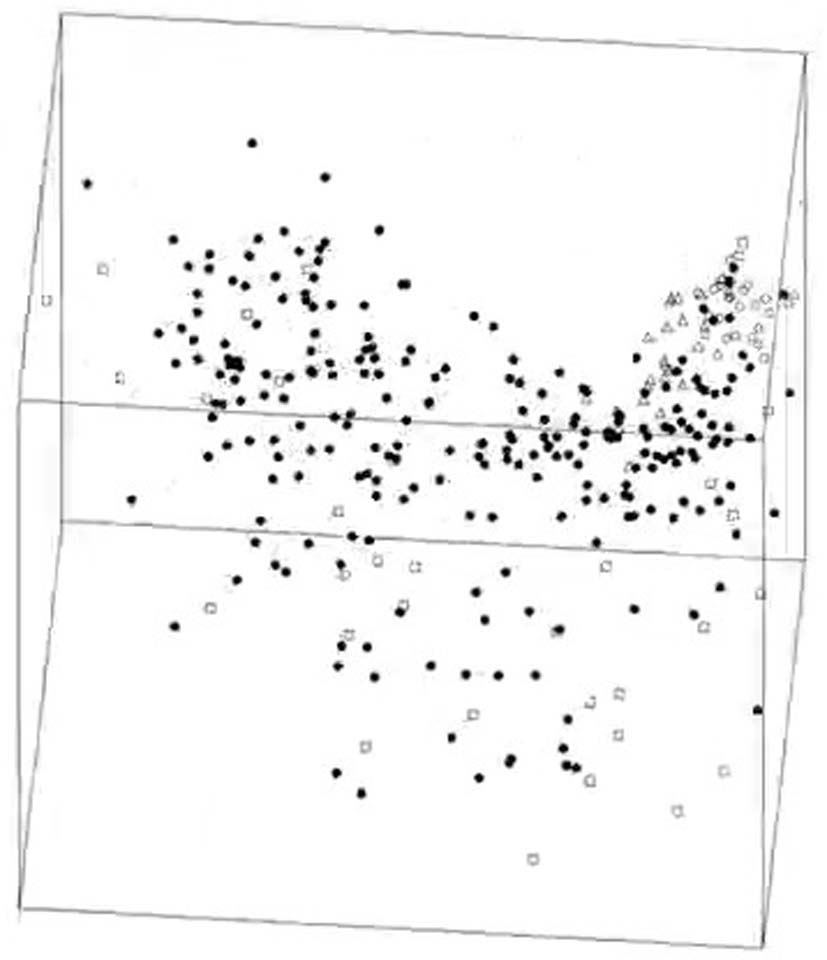

Supplement: Supplementary video — Animated 3D-plot of the principal component analysis (PCs 1–3). Symbols are equal to those used in Fig. 4. [file mmc2.jpg]
